# Beyond ADME: The Endogenous Functions of Drug Transporters and Its Impact on Human Disease

**DOI:** 10.3390/pharmaceutics17060685

**Published:** 2025-05-23

**Authors:** Christine Blaze, Yan Shu

**Affiliations:** Department of Pharmaceutical Sciences, School of Pharmacy, University of Maryland, Baltimore, MD 21201, USA; cblaze@umaryland.edu

**Keywords:** drug transporters, endogenous substrates, endogenous function, transporter-mediated pathophysiology, detoxification, immune modulation, metabolic regulation, barrier integrity, transporter dysregulation

## Abstract

Drug transporters are crucial for facilitating the distribution and elimination of drugs from the body, yet their broader physiological functions remain underexplored. Beyond drug handling, these transporters regulate key biological processes, including barrier integrity, metabolic homeostasis, detoxification, and immune response. Here we examine the endogenous roles of representative drug transporters along with their involvement in disease pathophysiology, particularly in neurological disorders, cancer, metabolic syndromes, kidney disease, and hepatic disorders. Given the complex interplay between transporters and various physiological processes, a comprehensive understanding of their roles beyond drug transport is imperative for advancing therapeutic strategies and fully grasping their impact on human health. By elucidating their mechanistic roles, researchers can refine drug development and identify novel therapeutic targets. This review underscores the need for continued research into transporters’ diverse functions and their potential as key modulators in disease prevention and treatment.

## 1. Introduction

Drug transporters are membrane proteins that regulate the movement of therapeutics across cell barriers. Through their regulation of intracellular and systemic drug concentrations, they directly impact therapeutic efficacy, drug interactions, and safety profiles [1,2,3,4,5]. Consequently, regulatory agencies, including the FDA, prioritize the study of drug transporters due to their role in determining drug pharmacokinetics and clinical outcomes [6,7,8].

Beyond their well understood function in drug transport, these membrane proteins ensure that essential biochemical processes proceed without disruption by facilitating the movement of endogenous molecules, including bile acids, hormones, and neurotransmitters, across biological membranes. Through this regulation, transporters maintain proper cellular function and physiological balance by controlling energy metabolism, signal transduction, and detoxification. Disruptions in transporter function are implicated in numerous pathological conditions, including metabolic syndromes, neurodegenerative disorders, and organ dysfunction.

This review provides a comprehensive analysis of the endogenous roles of nine FDA-prioritized drug transporters, detailing their functions beyond pharmacokinetics, including barrier maintenance, metabolic regulation, detoxification, and immune system modulation. Furthermore, it explores transporter dysfunction in disease states such as neurodegenerative disorders, cancer, metabolic syndromes, and organ failure, highlighting their implications for disease progression and treatment strategies. By linking transporter biology with disease pathology, this review aims to enhance our understanding of these proteins and their potential as therapeutic targets.

## 2. Overview of Drug Transporters

Among membrane transporters, drug transporters are notable due to their role in drug absorption, distribution, metabolism, and excretion (ADME) [9]. Their impact on pharmacokinetics and involvement in pathophysiological conditions makes them essential for understanding drug efficacy, toxicity, and drug–drug interactions [10]. Additionally, their endogenous functions, such as transporting hormones, bile acids, and metabolites essential for signaling pathways, are integral to maintaining physiological homeostasis [11,12,13]. Given this dual function, drug transporters are not only critical determinants of therapeutic outcomes but also valuable targets for drug development and potential biomarkers in clinical research. As a result, regulatory agencies, including the FDA and EMA, have placed significant emphasis on characterizing these transporters, leading to the development of guidelines as part of the Critical Path Initiative (CPT) to ensure their proper assessment in drug approval processes [14,15]. However, while the regulatory focus has been put on drug-transporting function, their endogenous roles remain underrepresented in research. This oversight is concerning, as understanding these transporters beyond their interactions with xenobiotics is crucial for uncovering novel therapeutic strategies, predicting off-target drug effects, and addressing potential metabolic dysregulations. Expanding research into their physiological roles is imperative to fully grasp their contributions in both health and disease.

**Figure 1:**
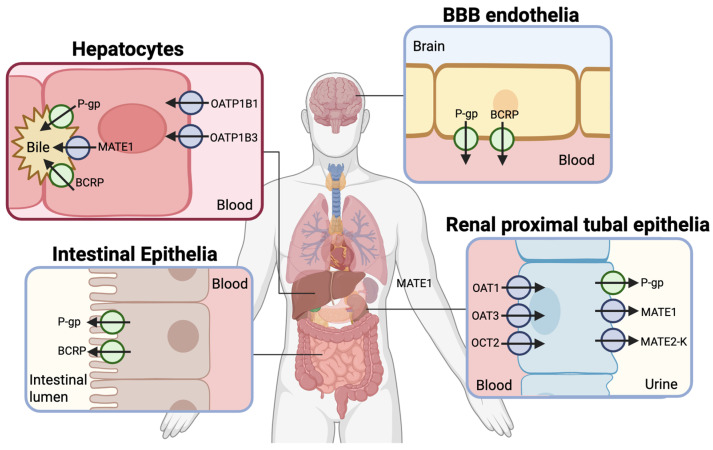
Drug transporter expression across tissues. The tissue-specific localization and directional activity-indicated by arrow direction-of key drug transporters in hepatocytes, renal proximal tubule epithelia, intestinal epithelia, and brain endothelial cells at the blood–brain barrier (BBB) are illustrated. ABC transporters (e.g., P-gp, BCRP) and SLC transporters (e.g., OATP1B1, OATP1B3, OAT1, OAT3, OCT2, MATE1, MATE2-K) mediate substrate movement across apical and basolateral membranes to regulate the distribution of drugs and endogenous metabolites between compartments such as blood, bile, urine, the intestinal lumen, and the brain.

To better understand these diverse roles, the broader classification of drug transporters and how their structural and functional differences influence both endogenous and drug-related transport mechanisms must be considered (Figure 1, Table 1). Drug transporters are categorized into two superfamilies: the ATP-binding cassette (ABC) transporters and the solute carrier (SLC) transporters. ABC transporters are active transport proteins powered by ATP hydrolysis to pump substrates across cellular membranes against their concentration gradients. They predominantly function as efflux pumps, protecting cells by exporting xenobiotics and endogenous substrates. Notable ABC drug transporters include P-glycoprotein (P-gp) and Breast Cancer Resistance Protein (BCRP) (Figure 1). P-gp is widely expressed in tissues such as the intestines, liver, kidneys, and the blood–brain barrier. The transporter is best known for its role in modulating intracellular drug accumulation [16]. Its overexpression is a major factor in multidrug resistance in cancer and influences the pharmacokinetics of various therapeutic agents. Beyond its role in drug transport, P-gp ensures the protection of sensitive tissues, such as the brain, by limiting the entry of potentially harmful substances. BCRP plays a similar protective role by effluxing drugs and metabolites across biological barriers. It is highly expressed in the placenta, where it prevents fetal exposure to xenobiotics. In addition, BCRP is expressed in the liver, intestine, and brain, where it contributes to resistance against chemotherapeutic drugs and facilitates the transport of dietary flavonoids and endogenous molecules such as urate.

SLC transporters primarily facilitate the uptake of various substrates through facilitated diffusion or secondary active transport, utilizing existing electrochemical gradients to drive the movement of molecules across membranes. These transporters are crucial for maintaining homeostasis and interorgan communication. Key SLC drug transporters include: Organic Anion Transporting Polypeptides (OATP1B1 and OATP1B3), Organic Anion Transporters (OAT1 and OAT3), Organic Cation Transporters (OCT2), and Multidrug and Toxin Extrusion Proteins (MATE1 and MATE2-K) (Figure 1).

OATP1B1 and OATP1B3 are relatively hepatocyte-specific transporters essential for the hepatic uptake of endogenous compounds such as bile acids, bilirubin conjugates, and coproporphyrins. They contribute to bile acid recycling and bilirubin clearance, with dysfunction leading to hyperbilirubinemia, as observed in Rotor syndrome [12,17]. Although they share overlapping substrate specificities, they differ in key functional properties: OATP1B3 exhibits a unique histidine-mediated, pH-dependent transport mechanism that has an enhanced uptake under acidic conditions, a feature absent in OATP1B1 [18]. Additionally, OATP1B3 alone mediates the hepatic uptake of certain peptide hormones such as cholecystokinin, suggesting functional diversification beyond canonical organic anion transport.

Human OAT1 and OAT3 are primarily expressed in the basolateral membrane of renal proximal tubule cells, where they mediate the uptake of a broad range of organic anions, including metabolic waste products and various medications. These transporters are essential for maintaining renal function and facilitating the clearance of endogenous compounds such as urate, prostaglandins, and bile acid derivatives—key molecules involved in purine metabolism, vascular regulation, and lipid homeostasis, respectively [19,20,21]. While both transporters contribute to renal clearance, OAT1 primarily favors smaller, redox-active anions such as dicarboxylates, supporting localized metabolic and redox processes in the proximal tubule. In contrast, OAT3 has a greater capacity for transporting structurally complex and conjugated metabolites, including glucuronidated compounds, and plays a broader role in systemic metabolite handling [20,22].

Like OAT1 and OAT3, human OCT2 is primarily expressed in the basolateral membrane of the renal proximal tubule cells in the kidneys. However, OCT2 mediates the uptake of organic cations from the blood for excretion. It plays a pivotal role in the renal clearance of drugs such as metformin and cisplatin and is implicated in drug-induced nephrotoxicity for certain drug classes. OCT2’s endogenous role in renal function is closely tied to maintaining systemic homeostasis. For example, it regulates the renal clearance of dopamine, an essential neurotransmitter that also functions as a signaling molecule in the kidney [23]. This process helps maintain physiological equilibrium and supports proper kidney-mediated signaling pathways.

MATE1 and MATE2-K are apical transporters in renal proximal tubular cells that function in sequence with basolateral OCTs to mediate the excretion of certain cations from blood to urine. While both contribute to renal clearance, MATE1 is also expressed in the liver, supporting a broader role in systemic excretion, whereas MATE2-K is kidney-specific, reflecting specialization in renal handling [24]. Although their substrates largely overlap, MATE1 displays broader specificity, including certain zwitterionic and anionic compounds not efficiently transported by MATE2-K—differences likely due to subtle variations in substrate binding [25]. These distinctions enable complementary handling of a wide range of endogenous metabolites, including bile acids, uremic toxins, and signaling molecules, thereby supporting physiological homeostasis and renal function [26,27].

**Table 1:** Summary of endogenous substrates by transporter.

Transporter	Gene	Endogenous Substrates	Primary Tissues	Citations
P-gp	*ABCB1*	Steroids (e.g., cortisol), neurotoxins (e.g., beta-amyloid), lipids (e.g., Phosphatidylcholine), endocannabinoids (e.g., anandamide)	Brain (BBB), intestine, liver, kidney	[28,29,30,31]
BCRP	*ABCG2*	Uric acid, bile acids (e.g., glycocholic acid), porphyrins (e.g., protoporphyrin IX), sulfated estrogens (e.g., estrone sulfate), neurotoxins (e.g., beta-amyloid)	Intestine, placenta, brain, liver	[32,33,34,35]
OATP1B1	*SLCO1B1*	Bilirubin, bile acids (e.g., taurocholate, glycochenodeoxycholate sulfate, glycodeoxycholic acid sulfate), hormones and steroid conjugates (e.g., estradiol-17β-glucuronide, Estrone-3-sulfate), Porphyrins (e.g., coproporphyrin I)	Liver	[36,37,38,39,40]
OATP1B3	*SLCO1B3*	Bilirubin, bile acids (e.g., taurocholate, glycochenodeoxycholate sulfate, glycodeoxycholic acid sulfate), hormones and steriod conjugates (e.g., estradiol-17β-glucuronide, estrone-3-sulfate), Porphyrins (e.g., protoporphyrin IX), Peptide hormones (e.g., cholecystokinin octapeptide (CCK-8))	Liver	[39,41,42,43]
OAT1	*SLC22A6*	Uremic toxins (e.g., indoxyl sulfate, p-cresyl sulfate), prostaglandins (e.g., PGE2), Tryptophan metabolites (e.g., kynurenine, xanthurenic acid), Vitamins (e.g., nicotinate (niacin), 4-pyridoxic acid (vitamin B6 metabolite), pantothenic acid), taurine, creatinine	Kidney	[44,45,46,47]
OAT3	*SLC22A8*	Uremic toxins (e.g., indoxyl sulfate, p-cresyl sulfate, CMPF, kynurenic acid), prostaglandins (e.g., PGE2), cyclic nucleotides (e.g., cAMP, cGMP), kynurenic acid, xanthurenic acid, estrone sulfatetaurine, *N*-acetylaspartate	Kidney	[20,47,48,49,50]
OCT2	*SLC22A2*	Uremic toxins (e.g., creatinine, guanidine, methylguanidine, guanidinosuccinic acid), Neurotransmitters and neuromodulators (e.g., dopamine, serotonin, histamine), Vitamins and derivatives (e.g., 1-methylnicotinamide), tryptophan	Kidney	[51,52,53,54]
MATE1	*SLC47A1*	Vitamins and cofactors (e.g., *N*-methylnicotinamide, thiamine, carnitine), Neurotransmitters and derivatives (e.g., histamine, serotonin), Organic cations and uremic solutes (e.g., creatinine, guanidine, TMAO), Anionic conjugates (e.g., estrone sulfate)	Kidney, liver	[24,53,55,56,57]
MATE2-K	*SLC47A2*	Uremic toxins and nitrogenous wastes (e.g., Creatinine, guanidine), Vitamin metabolites and cofactors (e.g., *N*-methylnicotinamide (NMN), thiamine (Vitamin B1), Steroid conjugates (Estrone sulfate)	Kidney	[24,55,56]

## 3. Endogenous Roles of Drug Transporters in Physiology

**Figure 2:**
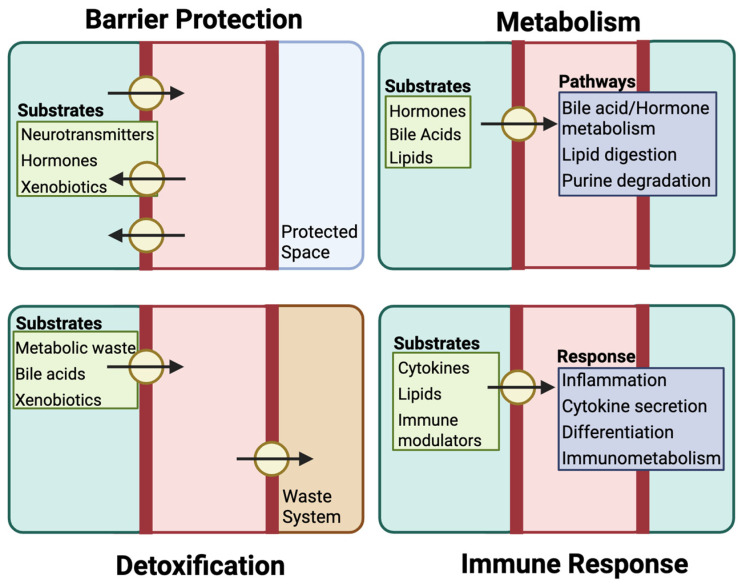
Physiological functions of key drug transporters. Drug transporter proteins, in addition to their function in pharmacokinetics, facilitating the maintenance of systemic and cellular homeostasis by controlling the movement of a wide range of substrates across cellular barriers. Through their efflux function, drug transporters support barrier protection by preventing the accumulation of compounds such as hormones or xenobiotics in sensitive tissues. Their regulation of intracellular substrate levels also shapes metabolic activity, influencing processes such as bile acid recycling, lipid processing, and purine turnover. In parallel, transporters work alongside metabolic enzymes in detoxification, eliminating waste products and reactive metabolites to reduce cellular stress and prevent toxicity. Additionally, these transporters help coordinate immune responses by modulating the availability of signaling molecules that affect inflammation, cytokine activity, and immune cell behavior.

**Figure 3:**
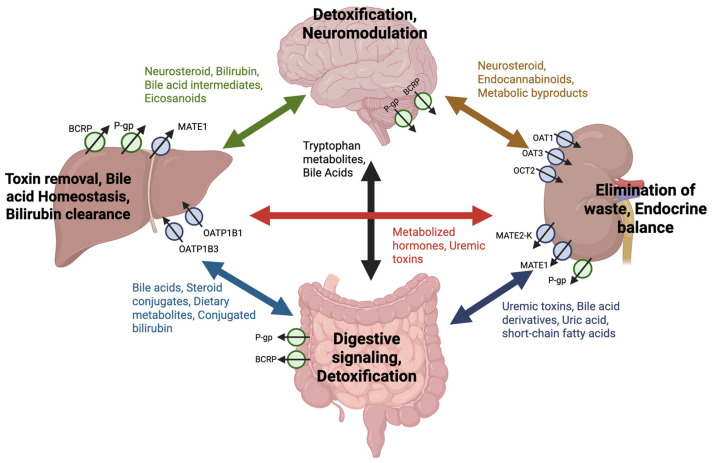
Inter-organ network of endogenous substrate transport by drug transporters. Drug transporters can mediate a bidirectional exchange of endogenous compounds between the brain, liver, kidney, and intestine, forming a dynamic inter-organ communication network. Transporters, including P-gp, BCRP, OAT1, OAT3, OCT2, MATE1, MATE2-K, OATP1B1, and OATP1B3, facilitate the movement of substrates such as bile acids, neurosteroids, uremic toxins, dietary metabolites, and metabolic byproducts. Arrows indicate where transporter-regulated uptake and efflux contributes to systemic homeostasis via the circulation of endogenous compounds between organs. The physiological functions supported by this exchange—such as detoxification, neuromodulation, bile acid homeostasis, digestive signaling, and endocrine balance—are labeled at each organ to contextualize the role of transporters in maintaining whole-body function through metabolite signaling and clearance.

The central function of transporter proteins is to maintain systemic and cellular homeostasis by controlling the movement of a wide range of substrates across cellular barriers. Consequently, transporters have many physiological roles including barrier protection, regulating metabolism, facilitating detoxification, and modulating immune responses (Figure 2). Beyond these central functions, transporters are also theorized to work with metabolizing enzymes and regulatory molecules to form inter-organ communication networks (Figure 3). This enables more precise regulation of metabolites and signaling molecules to better maintain systemic homeostasis, allowing tissues to monitor and respond to environmental and metabolic changes. This section provides an exploration of drug transporters in these endogenous roles and their contributions to health in human physiology.

### 3.1. Barrier Function and Protection

The role of drug transporters in barrier function and protection is primarily facilitated by efflux transporters, which act as gatekeepers to preserve the selective permeability of epithelial and endothelial barriers. These transporters are strategically expressed in barrier tissues, such as the blood–brain barrier (BBB), intestinal epithelium, and placenta, where they restrict the diffusion of potentially harmful compounds into sensitive tissues. This protective function has garnered significant attention, particularly as many studies focus on understanding how these transporters impede the entry of therapeutic agents into these tissues [58,59,60].

This protective function is exemplified by the BBB where several efflux transporters, including P-gp and BCRP, work in tandem to create a comprehensive defense mechanism [61]. This collaboration ensures redundancy and robustness in the BBB’s ability to limit the permeability of harmful substances. Together these transporters are responsible for preventing the entry of potentially neurotoxic compounds into the central nervous system. In addition to restricting the entry of drugs and toxins, transporters also mitigate the accumulation of reactive oxygen species and their precursors in the brain, helping mitigate oxidative stress in the central nervous system [62]. Furthermore, their role extends to the regulation of hormone permeability. P-gp, for example, has been shown to transport testosterone and androstenedione, contributing to the regulation of hormonal balance and potentially influencing neural function [11,63,64].

In the intestine, P-gp and other efflux transporters play a pivotal role in limiting the absorption of drugs and toxins. These transporters act as a first line of defense, reducing systemic exposure to potentially harmful ingested compounds by excreting them back into the intestinal lumen. However, this active efflux process significantly decreases drug bioavailability [65]. Additionally, they prevent the accumulation of endogenous compounds such as bile acids and conjugated metabolites that, at elevated levels, can become toxic to the intestinal epithelium. This protective mechanism prevents the continuous exposure of the intestinal epithelium to dietary xenobiotics, drugs, and microbial toxins, which pose a significant threat to its structural and functional integrity [66].

In the placenta, P-gp and BCRP serve protective roles by restricting fetal exposure to potentially harmful substances in the maternal bloodstream. These transporters create an effective barrier to drugs, toxins, and endogenous compounds that could otherwise adversely affect fetal development. They maintain fetal safety by regulating the maternal-fetal exchange of substrates [67,68]. Additionally, their expression adapts throughout pregnancy to meet the changing needs of the developing fetus, further underscoring their dynamic protective roles [69].

### 3.2. Metabolic Regulation

Drug transporters regulate endogenous metabolites by mediating their movement across cellular and organ barriers. Their broad substrate specificity enables them to transport a diverse array of metabolites, including bile acids, steroid hormones, uremic toxins, and lipids. By ensuring substrate flux and systemic balance, these transporters influence numerous physiological processes and metabolic pathways critical for homeostasis.

As described above, transporters in the placenta shield the fetus from potentially harmful substances. Moreover, transporters including OATPs and P-gp facilitate the exchange of key metabolites between maternal and fetal systems. OATP2B1 mediates the transport of steroid conjugates and bile acids, contributing to fetal development and placental metabolic stability [70]. Similarly, P-gp not only protects the fetus by effluxing xenobiotics but also regulates the levels of steroid hormones and lipids within the placental barrier, ensuring appropriate substrate availability for fetal growth [71,72,73]. Dysregulation of these transporters can impair placental function, resulting in complications such as fetal growth restriction or maternal metabolic imbalances [74].

After birth, drug transporters in the mammary gland help regulate the nutritional composition of breast milk, supporting neonatal physiology. BCRP, which is highly upregulated during lactation, actively secretes substrates such as riboflavin and uric acid, modulating the milk’s micronutrient profile and contributing to early metabolic maturation. Functional studies in BCRP-knockout mice demonstrate significantly reduced concentrations of these compounds, confirming the transporter’s role in maintaining milk composition [75]. Additionally, OCT1 and OCT2 have been implicated in the selective transfer of cationic metabolites into breast milk, enhancing its nutrient content [76]. This transporter-mediated exchange extends maternal metabolic regulation beyond pregnancy, helping support the infant’s developmental and physiological needs.

Beyond maternal-infant exchange, drug transporters demonstrate their pivotal role in regulating metabolites across various organ systems. In the liver, OATP1B1 and OATP1B3 mediate the uptake of bile acids, bilirubin, and hormones, ensuring efficient metabolic processing and detoxification. Dysregulation of these hepatic transporters can lead to conditions like cholestasis or hyperbilirubinemia, with broader consequences for lipid metabolism and cardiovascular health [77]. In the kidney, OAT1, OAT3, and OCT2 facilitate the excretion of uremic toxins such as indoxyl sulfate and uric acid, maintaining nitrogen balance and systemic metabolic stability [46,78,79]. Impaired renal transporter function can disrupt the gut-liver-kidney axis, contributing to systemic inflammation, accumulation of toxins, and downstream metabolic dysregulation [20,80].

Transporters also play essential roles in lipid and energy metabolism. In the intestine, transporters regulate the movement of bile acids, which are not only essential for lipid digestion and absorption, but also function as signaling molecules critical for energy homeostasis [81]. P-gp actively redistributes cholesterol within cell membranes, indirectly modulating cellular signaling and metabolic activity [82]. Moreover, MATE1 has been shown to modulate lipid composition by reducing the production of polyunsaturated fatty acid cholesterol esters, protecting cells from ferroptosis and highlighting the broader implications of transporters in maintaining metabolic balance [26]. These functions, combined with their role in regulating endogenous metabolites, underscore the diverse and systemic impacts of transporter-mediated metabolic regulation.

Similarly, transporters ensure cell viability and functionality by facilitating the uptake of essential nutrients and metabolites needed for energy production, biosynthesis, and signaling pathways that drive immune responses [83]. For instance, OATs in T lymphocytes mediate the transport of lactate, a metabolite that supports glycolysis, which is critical for the rapid proliferation and activation of these cells during immune challenges [84]. By providing a constant supply of these vital molecules, transporters sustain the metabolic demands of immune cells, enabling them to adapt to and effectively combat pathogens or inflammation.

### 3.3. Detoxification

Detoxification is another essential role of drug transporters, involving the clearance of potentially harmful substances. Unlike barrier protection, which limits the distribution of toxins into sensitive tissues, detoxification removes compounds circulating in extracellular fluids or generated within cells to prevent local and systemic toxicity. Efflux transporters such as MATEs and BCRP expel toxic compounds and drug metabolites from cells, while uptake transporters like OAT1 and OAT3 facilitate the import of toxins for intracellular processing and subsequent excretion. This interplay of transporter activity highlights the importance of cellular detoxification not only in protecting individual cells but also in maintaining systemic homeostasis.

Drug transporters including OCTs, MATEs, OATs, and P-gp are all expressed in renal epithelial cells, where they coordinate toxin uptake and elimination. For example, OCT2 facilitates the uptake of cationic metabolites, such as creatinine, into renal cells, while MATE1 and MATE2-K export these metabolites into urine, completing the detoxification process [79]. Similarly, OAT1 and OAT3 handle a diverse array of organic anions, including oxidized fatty acids and protein-bound toxins such as indoxyl sulfate. Their ability to import these metabolites into renal epithelial cells facilitates further metabolic processing or excretion [21,85,86]. These coordinated activities prevent intracellular toxin accumulation, maintain cellular homeostasis, and support toxin clearance. Disruptions in these transporters’ functions can lead to the buildup of uremic toxins, exacerbating conditions such as chronic kidney disease (CKD) and promoting systemic complications [87,88].

While renal epithelial cells specialize in clearing toxins through urinary excretion, hepatocytes provide complementary detoxification through bile-mediated elimination. Many of the same transporters, including P-gp, BCRP, and MATE1, are active in hepatocytes, where they coordinate with uptake transporters such as OATP1B1 and OATP1B3. The uptake transporters import organic anions, including bile salts, conjugated bilirubin, and other metabolites, into hepatocytes for processing and excretion. Efflux transporters like BCRP and P-gp play essential roles in exporting processed toxins into bile canaliculi, preventing intracellular accumulation and ensuring efficient excretion. Dysregulation of these processes due to transporter inhibition, genetic polymorphisms, or disease can lead to hepatocyte dysfunction, toxic metabolite buildup, and pathologies such as cholestasis and drug-induced liver injury [89]. However, it should be noted that although this review exemplifies drug transporter-mediated detoxification in hepatocytes and renal epithelial cells, these activities also occur in other cells and tissues expressing drug transporters, including the BBB, placenta, and intestinal epithelia [90,91,92]. By efficiently managing the import, processing, and export of toxins, drug transporters exemplify the sophisticated cellular mechanisms underlying detoxification, thus maintaining cellular integrity, preventing local and consequently systematic toxicity.

### 3.4. Immune Modulation

Drug transporters regulate immune function by controlling the movement of signaling molecules across cellular membranes. By modulating intracellular and extracellular concentrations of cytokines, lipids, and other immunomodulatory compounds, these transporters ensure proper immune cell activation and communication, maintaining immune homeostasis and supporting adaptive responses during pathogenic or inflammatory challenges. Dysregulation of transporter expression or function can lead to immune imbalances that contribute to autoimmune disorders and chronic inflammation. Of the drug transporters implicated in immune regulation, P-gp is by far the most extensively characterized, offering a foundational model for how transporter activity can shape immune signaling. In contrast, the immunological functions of many other transporters remain poorly understood and warrant further investigation.

OATP family members, such as OATP1B3, exemplify the role of uptake transporters in immune modulation, facilitating the import of bile acids that act as signaling molecules to influence T cell responses and systemic inflammation [41]. On the efflux side, transporters like P-gp help maintain immune equilibrium by exporting immunologically active compounds, including endocannabinoids, cytokines, and xenobiotics, preventing excessive local immune activation [31,93]. Their function is not limited to inflammatory contexts; even under homeostatic conditions, P-gp contributes to baseline immune surveillance. For instance, in healthy nasal epithelial cells, P-gp regulates intracellular cytokine levels and inflammatory signaling, suggesting that it plays a constitutive role in immune tone regulation [94].

The expression and function of these transporters are tailored to the immune needs of specific organs as well. In the brain, P-gp at the blood–brain barrier helps limit neuroinflammation by reducing pro-inflammatory mediator accumulation [95]. In the kidney, P-gp is upregulated in macrophages in response to damage signals like high mobility group box 1 (HMGB1), altering local immune dynamics [96]. Similarly, in the lungs, P-gp, MATE1, and Organic Cation Transporter Novel Type 1 and 2 (OCTN1 and OCTN2) are differentially expressed in health and COPD, reflecting shifts in immune regulation by these transporters under chronic inflammatory conditions [97].

Inflammatory signaling also modulates transporter expression. In peripheral immune cells, chronic inflammation upregulates P-gp through STAT3 and NF-κB pathways, suggesting a feedback mechanism where inflammation drives transporter activity to control the immune environment [98]. However, this response can be maladaptive; in acute kidney injury, HMGB1-induced P-gp upregulation reshapes macrophage function, sometimes exacerbating inflammation [99]. Thus, transporters are not just responders to inflammation—they can actively shape its trajectory.

Experimental models reinforce the role of transporters in immune regulation. Mice lacking mdr1a, which encodes P-gp, spontaneously develop colitis resembling human inflammatory bowel disease, illustrating the consequences of transporter dysfunction for epithelial barrier integrity and immune balance [100,101]. In humans, altered transporter expression in diseases like COPD further underscores their role in immune dysregulation [97]. ABC transporters more broadly have been linked to autoimmune diseases, where their dysfunction leads to persistent immune activation and skewed cytokine responses [93,102].

Altogether, drug transporters are integral to the orchestration of immune responses. They function not only as regulators of molecular flux but as active participants in immune surveillance, inflammation resolution, and disease pathogenesis. While P-gp’s role in immune modulation has been widely studied, the immunological roles of other transporters remain comparatively less characterized. Expanding our understanding of these lesser-studied transporters could reveal new insights into immune regulation and identify novel targets for treating inflammatory and immune diseases.

## 4. Dysregulation of Drug Transporters in Disease Pathophysiology

**Figure 4:**
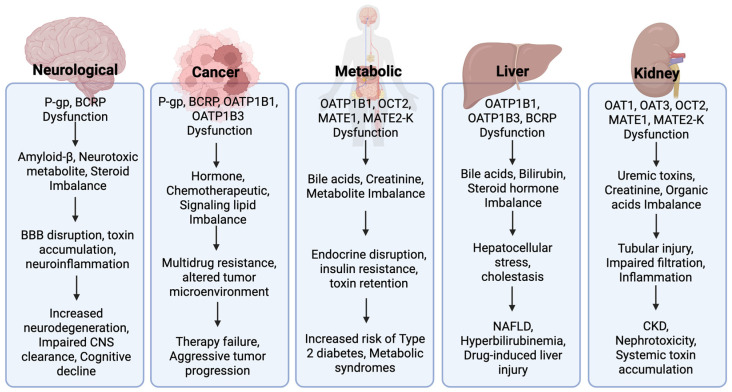
Mechanistic flow of transporter dysfunction in disease pathophysiology. Dysfunction of key drug transporters contributes to the pathophysiology across neurological, oncologic, metabolic, hepatic, and renal contexts. For each category, transporter dysfunction (e.g., P-gp, BCRP, OATP1B1/B3, OAT1/3, OCT2, MATE1/2-K) leads to disrupted handling of endogenous substrates—including bile acids, neurotoxic metabolites, creatinine, and steroid hormones—resulting in physiological disturbances such as inflammation, barrier disruption, or metabolic imbalance. These disruptions are associated with adverse outcomes such as neurodegeneration, drug resistance, insulin resistance, cholestasis, and chronic kidney disease.

Drug transporters are not only necessary to maintain physiological equilibrium under normal conditions, but their dysregulation can disrupt tissue function and inter-organ communication (Figure 4). Altered transporter expression or activity has been observed in a range of pathologies, including cancer, kidney disease, liver dysfunction, and neurological disorders, where they contribute to disease progression by perturbing the balance of endogenous metabolites, signaling molecules, and toxins. Understanding these changes is essential for unraveling how transporter dysfunction intersects with disease mechanisms and may reveal new opportunities for therapeutic intervention or biomarker development.

### 4.1. Neurological Disorders

The BBB maintains the central nervous system’s (CNS) microenvironment by regulating the movement of substances between the bloodstream and the brain. BBB dysfunction, including transporter dysregulation, has been increasingly implicated in the progression of neurodegenerative disorders such as Alzheimer’s disease (AD), Parkinson’s disease (PD), Huntington’s disease (HD), and multiple sclerosis (MS), where impaired efflux capacity facilitates the accumulation of toxic species and exacerbates neuroinflammation and protein aggregation [103,104,105]. One example of this phenomenon is seen in AD, where transporter impairment contributes directly to the accumulation of neurotoxic amyloid-β (Aβ) peptides.

Aβ accumulation is a hallmark of AD and a major driver of neurodegeneration. P-gp and BCRP are involved in clearing Aβ from the brain. Both transporters actively export Aβ peptides into the bloodstream, helping prevent their buildup in neural tissues [35,106]. Studies suggest that reduced expression or activity of P-gp and BCRP contributes to impaired clearance of Aβ and is associated with increased cerebral Aβ accumulation in AD. In vivo experiments using transgenic mouse models demonstrate that inhibition or genetic loss of P-gp alone results in a 2- to 3-fold increase in brain Aβ levels, underscoring its neuroprotective role, while BCRP acts synergistically by facilitating complementary Aβ efflux [29,107]. These findings have motivated therapeutic strategies aimed at enhancing P-gp and BCRP function [108,109].

In PD, polymorphisms in the *ABCB1* gene (encoding P-gp) influence transporter efficiency and may alter susceptibility to neurotoxin-induced neuronal damage. For instance, the C3435T variant has been shown to reduce transporter activity, increasing toxin accumulation in dopaminergic neurons [110]. However, genetic effects vary across populations, with haplotypes such as 2677T-3435T offering protective benefits [111].

Similar patterns of transporter involvement have been observed in other neurodegenerative disorders. In HD, decreased P-gp function has been associated with impaired clearance of mutant huntingtin protein aggregates, contributing to neuronal dysfunction and disease progression [112]. In MS, P-gp and BCRP are involved in modulating immune cell trafficking across the BBB. Dysregulation of these transporters facilitates peripheral immune infiltration into the CNS, promoting neuroinflammation and lesion development [113].

Targeting transporters like P-gp and BCRP is now being considered a potential therapeutic approach for neurodegenerative diseases. Advancements in nanoparticle delivery systems and proteasome inhibitors have highlighted the feasibility of modulating transporter activity to improve amyloid clearance and reduce oxidative stress [108,109]. Furthermore, genetic profiling offers a pathway to personalized medicine, tailoring interventions to individual transporter functionality. However, challenges remain, particularly in ensuring targeted therapies do not disrupt physiological processes. Continued interdisciplinary research and clinical trials are essential to translating these strategies into effective treatments for conditions such as AD, PD, HD, and MS.

### 4.2. Cancer

Drug transporters are well recognized for their role in drug resistance in cancer therapy. However, viewing them through the lens of endogenous function reveals their broader influence on tumor biology, progression, and immune interactions beyond mediating drug ADME. Somatic mutations and transcriptomic alterations in drug transporter genes provide compelling evidence of their involvement in cancer cell survival [114]. P-gp, BCRP, and OATPs regulate the movement of endogenous molecules such as hormones and bile acids, which are essential for sustaining tumor growth. The altered expression of these transporters in cancer suggests that tumors may exploit them to enhance metabolic efficiency, support proliferation, and evade cell death mechanisms.

Epigenetic modifications, particularly DNA methylation, play a significant role in regulating transporter gene expression. Methylation-driven dysregulation of transporters, particularly P-gp and BCRP, enables tumors to fine-tune their metabolic processes, ensuring optimal intracellular conditions for survival [115]. The ability of tumors to dynamically adjust transporter expression in response to their metabolic needs highlights a critical, but often overlooked, endogenous function of these proteins in cancer pathology. Similarly, OATPs, which mediate the uptake of various endogenous signaling molecules, display tumor-specific expression patterns [116]. In certain cancers, these transporters facilitate the import of growth-promoting substrates such as steroid hormones, further driving tumor proliferation. For example, OATP1B3 is overexpressed in prostate tumors and mediates the uptake of testosterone and its precursors, contributing to elevated intratumoral androgen levels and tumor progression [117,118]. In these contexts, OATPs may be repurposed to enhance malignant potential. Understanding how tumors exploit OATP transporters could pave a novel way for therapeutic strategies aimed at disrupting these metabolic dependencies.

Beyond metabolism, drug transporters exert significant effects on the tumor immune microenvironment (TIME), influencing immune modulation, stress responses, and interactions with stromal and immune cells [119]. ABC transporters, including P-gp and BCRP, have been shown to modulate TIME by regulating extracellular concentrations of cytokines, chemokines, and metabolites. These transporters are overexpressed in tumors such as colon cancer and leukemia, where they efflux immunosuppressive molecules like prostaglandins and leukotrienes [120,121]. P-gp overexpression has also been implicated in dampening immune surveillance, potentially by limiting dendritic and T cell infiltration and contributing to therapy resistance [122]. BCRP is similarly upregulated in hypoxic, stem-like tumor regions that are poorly infiltrated by immune cells, suggesting a role in sustaining immune-evasive niches [123]. By altering the extracellular balance of cytokines and metabolites, these transporters can reprogram the TIME to suppress immune recognition and response. Some tumors exploit this mechanism to evade immune detection. Therapeutically targeting these transporters may help restore immune surveillance and improve the efficacy of cancer immunotherapies.

The broader influence of drug transporters on tumor progression and therapy resistance indicates their potential as therapeutic targets. Modulating transporter activity offers potential strategies not only for overcoming drug resistance but also for altering the tumor’s pathophysiological landscape to limit disease progression. Moreover, the adaptability of tumors in modulating transporter expression in response to metabolic and immune pressures underscores the need for dynamic therapeutic strategies. Future research should explore how modulating transporter function can disrupt tumor survival pathways, offering new avenues for therapeutic intervention in cancer treatment.

### 4.3. Metabolic Disorders

As previously discussed, drug transporters maintain metabolic homeostasis by regulating the uptake and excretion of key metabolites involved in glucose, lipid, and bile acid balance. Alterations in transporter function can disrupt these tightly regulated processes, leading to metabolic dysregulation and contributing to disease pathology. Dysregulation of key drug transporters can have profound implications for insulin resistance, glucose homeostasis, and drug metabolism in diabetes. For example, the genetic variant T201M of OCT2, which plays a central role in metformin clearance, has been linked to increased insulin resistance in patients with Type 2 Diabetes (T2D) [124]. Individuals carrying this variant exhibit higher fasting glucose and homeostatic model assessment of insulin resistance levels, indicating that altered OCT2 function may affect metformin’s efficacy and the body’s ability to regulate glucose. Beyond its renal role, OCT2 is also expressed in pancreatic β-cells, where its expression is upregulated under high glucose conditions, suggesting a potential role in insulin secretion and β-cell function [125].

Similarly, OATP1B1 and OATP1B3 have emerged as important regulators of glucose metabolism through their role in hepatic thyroid hormone uptake. These transporters facilitate the entry of thyroid hormones into hepatocytes, enabling activation of thyroid hormone receptors (TRs) that regulate genes essential to glucose homeostasis. In *Slco1b2* knockout mice, the murine ortholog functionally analogous to human OATP1B1 and OATP1B3, reduced hepatic thyroid hormone availability led to impaired glucose clearance, decreased hepatic glucose uptake, and downregulation of TR target genes such as GLUT2 and PEPCK, both key players in glucose transport and gluconeogenesis, suggesting that genetic variation in transporter function may contribute to hepatic insulin resistance and broader metabolic dysfunction [126].

Beyond genetic variation in transporter genes, diabetes itself alters drug transporter function in a tissue-specific manner, affecting drug distribution and efficacy. In streptozotocin-induced diabetic rats, P-gp expression and function were significantly impaired, altering the pharmacokinetics of various drugs and potentially exacerbating diabetes-related complications [127]. In vitro studies using HepG2 cells further show that insulin resistance activates the Protein kinase R-like ER kinase (PERK) signaling pathway, which upregulates P-gp, reduces intracellular drug accumulation, and likely makes diabetic patients more resistant to pharmacological interventions [128].

The impact of drug transporters on metabolic disorders is not diabetes specific, it extends to other metabolic disorders including obesity and dyslipidemia. OATP1B1 and OATP1B3 clear endogenous lipids, bile acids, and cholesterol metabolites, as well as mediating statin uptake for lipid-lowering therapy. Polymorphisms in OATP1B1 (OATP1B1*5 and OATP1B1*15) reduce transporter function, leading to higher systemic drug exposure, increased risk of statin-induced myopathy, and altered lipid metabolism, potentially worsening dyslipidemia [129]. Additionally, OATP1B3’s role in bile acid transport influences lipid and cholesterol metabolism. Downregulation of OATP1B3 is commonly observed in obesity-associated liver dysfunction and cholestasis. In these cases, the decreased OATP1B3 expression leads to reduced hepatic uptake of conjugated bile acids, impairing bile acid recycling and cholesterol clearance [41]. This disruption contributes to dyslipidemia as well, as bile acids regulate Low-Density Lipoprotein cholesterol levels and lipid metabolism [130].

Beyond its hepatic transport function, BCRP plays a key role in lipid metabolism and intestinal drug efflux. Obesity-induced inflammation has been reported to disrupt BCRP function, particularly in the intestine, leading to compromised drug efflux, increased gut permeability, and altered cholesterol handling. Studies have shown that intestinal BCRP dysfunction in obesity results from reduced Janus kinase 3 (JAK3)-mediated phosphorylation, which is essential for BCRP localization and activity, leading to increased drug retention and dysregulated gut-liver communication [131]. This dysfunction not only affects drug absorption but also exacerbates metabolic endotoxemia and chronic low-grade inflammation, further aggravating obesity-related metabolic disorders. The interplay between BCRP and lipid metabolism is also evident in dyslipidemia, where altered transporter expression affects the enterohepatic circulation of bile acids and cholesterol-derived metabolites [129].

Thus, the endogenous function of drug transporters can profoundly impact metabolic homeostasis, contributing to systemic imbalances that influence disease progression and therapeutic efficacy, promoting the development and progression of metabolic disorders such as diabetes, obesity, and dyslipidemia. These metabolic disturbances often have significant consequences for kidney and liver function, as both organs depend on transporter-mediated processes for waste elimination, drug clearance, and overall metabolic regulation. Impaired transporter activity in these organs can exacerbate disease pathology, reinforcing the intricate link between metabolic disorders and organ dysfunction.

### 4.4. Kidney Disease

Given their role in metabolic regulation and detoxification, drug transporters can influence the onset and progression of various renal diseases. Dysfunctional transporter activity disrupts the clearance of metabolic waste products, uremic toxins, and xenobiotics, contributing to pathological processes in both acute kidney injury (AKI) and CKD. In the proximal tubule, OAT1 and OAT3 facilitate the excretion of key solutes such as indoxyl sulfate, p-cresol sulfate, kynurenine, and urate [46]. These metabolites become cytotoxic when retained, driving oxidative stress and inflammation. Studies in Oat1 and Oat3 deficient mice show elevated plasma levels of uremic toxins, correlating with glomerular damage, tubular injury, and fibrosis in both progressive CKD and AKI models [132]. These findings suggest that OAT dysfunction is not merely a consequence of kidney injury but may actively contribute to its progression. Moreover, functional impairment of OAT1 and OAT3 in CKD progresses more rapidly than glomerular filtration loss, suggesting that transporter dysfunction may contribute to renal decline beyond filtration impairment [133].

MATE1, which facilitates the excretion of cationic solutes into the tubular lumen, plays a protective role in the kidney, particularly during injury. During CKD progression and toxin-induced renal injury, MATE1 expression is significantly reduced, leading to the accumulation of solutes such as creatinine and trimethylamine-*N*-oxide (TMAO), which contribute to nephrotoxicity and exacerbate renal damage [57,134,135]. In cisplatin-induced AKI mouse models, MATE1 deficiency increases the renal burden of toxic metabolites and worsens tubular injury [134]. Given its role in creatinine transport, MATE1 dysfunction may also interfere with clinical assessment of renal function, particularly in acute or fluctuating disease states. Genetic variants in *SLC47A1* (MATE1) have been associated with elevated serum creatinine levels that are disproportionate to actual glomerular filtration, raising concerns about the reliability of creatinine as a renal biomarker [136,137].

OCT2, which works in coordination with MATE1 to regulate renal cation homeostasis, is similarly vulnerable to suppression in disease. Inflammatory signals, including NF-κB and TNF-α, downregulate OCT2 expression in both AKI and CKD [138]. OCT2 activity is further inhibited by accumulating uremic toxins that act as endogenous inhibitors [139,140]. This disruption impairs cation handling and intensifies metabolic stress. Concurrently, fibrosis-associated changes in transporter expression reduce the kidney’s capacity to adapt to injury in both acute and chronic settings [141]. Emerging evidence also implicates transporter dysregulation in tubulointerstitial fibrosis, a hallmark of progressive renal decline. In fibrotic kidneys, OCT2 and MATE1 expression inversely correlate with serum creatinine levels and fibrosis severity, suggesting that their reduced expression may contribute to structural remodeling and loss of function. In contrast, P-gp expression appears upregulated in fibrosis, pointing to complex and possibly compensatory roles for individual transporters within the fibrotic milieu [141]. These findings underscore the broader involvement of transporter networks not only in solute clearance but also in the pathogenesis of structural kidney damage.

P-gp expression is altered in renal injury, although it appears less sensitive to inflammatory downregulation than OCT2 or MATE1 [142]. When P-gp function is impaired, hydrophobic metabolites and nephrotoxic compounds can accumulate, promoting lipid dysregulation and proximal tubule injury [143]. This mechanism likely contributes to both acute and chronic renal damage as well. Beyond metabolite accumulation, renal transporter dysfunction alters drug disposition, increasing systemic exposure and toxicity risk. In both acute and chronic renal injury, declining OAT1 and OAT3 function reduces anionic drug clearance, while impaired MATE1 and P-gp activity elevates plasma levels of cationic and hydrophobic drugs, respectively [144,145]. These changes may necessitate careful dose adjustment to maintain therapeutic efficacy and avoid further nephrotoxicity.

Whether in AKI, CKD, or other renal pathologies, transporter dysregulation contributes to impaired toxin clearance and disease progression. Transporter expression is shaped by inflammation, fibrosis, and systemic metabolic disturbances, indicating that these proteins are not just passive drug carriers but dynamic participants in kidney pathophysiology. A broader understanding of transporter involvement across the renal disease spectra is necessary for improving diagnostics, optimizing treatment regimens, and identifying novel targets to preserve renal function.

### 4.5. Hepatic Conditions

Hepatic transporters maintain physiological homeostasis by regulating the uptake and excretion of bile acids, endogenous metabolites, and xenobiotics. Their coordinated function supports detoxification, metabolic balance, and hepatocellular integrity. However, during liver injury the expression and activity of these transporters become dysregulated, disrupting solute handling and contributing to hepatocellular injury, inflammation, and fibrosis.

Bile acid dysregulation represents a central pathological feature of liver disease. Under normal conditions, hepatocytes import bile acids via uptake transporters such as OATP1B1 and OATP1B3 and excrete them into bile through canalicular efflux proteins like BCRP and MATE1. This cycle is tightly regulated to prevent intracellular bile acid accumulation, which can trigger oxidative stress, mitochondrial dysfunction, and inflammatory signaling. In cholestatic and fibrotic liver diseases, such as primary biliary cholangitis (PBC) and metabolic associated steatohepatitis (MASH), the expression of these bile acid transporters is significantly downregulated. In hepatitis C–associated liver injury, OATP1B3 expression is markedly reduced, impairing hepatic bile acid clearance and leading to their retention, while BCRP levels are elevated, potentially reflecting an adaptive but insufficient attempt to enhance bile acid efflux [146,147]. The resulting bile acid accumulation drives cytotoxicity that contributes directly to hepatocyte injury and disease progression [89,148,149,150].

Beyond bile acids, hepatic transporters are responsible for clearing a wide range of endogenous metabolites including bilirubin and sulfated steroid hormones, whose uptake is mediated by OATP1B1 and OATP1B3. Clinical studies have reported suppression of OATP1B expression in fibrotic liver tissue [151]. Given their role in transporting key endogenous substrates, such downregulation may contribute to hepatic solute retention and metabolic stress, promoting oxidative stress, altered redox balance, and impaired cellular metabolism [150,152]. In parallel, suppression of P-gp reduces the efflux of hydrophobic xenobiotics and metabolites, resulting in their intracellular retention and contributing to mitochondrial dysfunction and sustained hepatocellular stress [153].

Transporter dysfunction also plays a role in hepatic fibrosis. Persistent accumulation of bile acids, xenobiotics, and endogenous solutes activate pro-inflammatory and pro-fibrotic pathways. Downregulation of OATP1B1, OATP1B3, and BCRP reduces the liver’s ability to clear harmful metabolites. This impaired clearance elevates cytokine signaling and hepatic stellate cell activation [154]. These findings suggest that transporter impairment is not merely a consequence of liver fibrosis, but a driver of its progression.

Hepatic transporters are also involved in lipid metabolism. In MAFLD and MASH, P-gp downregulation is associated with increased hepatic lipid retention and metabolic stress [155]. OATP1B1 and OATP1B3 also facilitate the uptake of lipid-soluble molecules. Their dysfunction may contribute to lipotoxicity and oxidative injury by disrupting lipid homeostasis [149]. Moreover, impaired bile acid transport through BCRP further disturbs enterohepatic signaling and bile acid–mediated lipid regulation, compounding metabolic dysfunction in steatotic liver disease [156].

Transporter dysregulation is also a factor in drug-induced liver injury (DILI). Reduced OATP1B1 and OATP1B3 expression limits hepatic drug uptake, increasing systemic drug exposure and prolonging circulation of hepatotoxic compounds [89]. Impaired function of BCRP and MRP2 diminishes biliary drug excretion, while P-gp suppression facilitates accumulation of hydrophobic drugs within hepatocytes [152,156]. These disruptions increase the liver’s vulnerability to xenobiotic stress and exemplify the importance of transporter function in guiding safe pharmacotherapy in liver-impaired individuals. Pharmacoproteomic analyses of human liver biopsies have reported altered transporter expression in patients with liver disease, which may explain observed changes in drug pharmacokinetics and underscores the clinical relevance of transporter profiling in DILI risk assessment and therapeutic management [157].

Altogether, hepatic transporters maintain liver function by regulating a broad spectrum of endobiotic and xenobiotic substrates. Their dysfunction contributes to the pathogenesis of hepatic conditions through bile acid toxicity, oxidative stress, inflammation, metabolic imbalance, fibrosis, and impaired drug clearance. Targeting transporter function offers a promising therapeutic strategy, but such interventions must be carefully balanced to preserve essential detoxification and metabolic processes. As research progresses, integrating transporter-focused approaches with emerging tools such as omics profiling, spatial transcriptomics, and advanced liver models may unlock new opportunities for precise therapeutic strategies for hepatic conditions.

## 5. Research Gaps and Challenges

Understanding of the endogenous functions of drug transporters remains relatively limited due to a historical emphasis on their roles in drug disposition rather than their physiological and pathological significance. Consequently, their precise contributions to homeostasis and disease progression remain underexplored [158,159,160]. The limited functional characterization of these transporters impedes the development of targeted therapeutic strategies that leverage transporter biology for disease treatment [161]. Advancing this field will require addressing the biological and methodological complexities that obscure transporter function, including context-dependent expression, compensatory network effects, and the limitations of current laboratory and clinical tools.

A key difficulty in studying endogenous transporter function arises from the overlap in substrate specificity among different transporters. Many transporters share common endogenous substrates, complicating efforts to isolate individual contributions to physiological processes [24,159,162]. This functional redundancy further challenges characterization, as the disruption of one transporter frequently triggers compensatory mechanisms for others, confounding experimental interpretations [163]. Additionally, transporter expression is highly variable across tissues and developmental stages. Tissue-specific regulation, influenced by genetic, epigenetic, and environmental factors, further complicates functional analysis [164,165]. Developmental changes in transporter expression make it particularly difficult to extrapolate findings across different life stages, hindering the establishment of consistent functional roles across biological contexts [4,166].

Most research on drug transporters has focused on their hepatic and renal functions, leaving substantial knowledge gaps regarding their roles in less-studied tissues such as the placenta, adipose tissue, muscle, immune cells, and the brain. Despite their importance in systemic physiology, experimental approaches to studying transporters in these non-traditional sites remain underdeveloped. Moreover, existing experimental models have significant limitations. In vitro cell culture systems lack the physiological complexity necessary to fully capture transporter functions within the human body [167,168,169]. While knockout mouse models provide useful insights, they often fail to accurately replicate human transporter function due to species differences, thereby limiting translational relevance [170,171]. Furthermore, the absence of suitable human models for organ-specific transporter studies remains a significant obstacle to the clinical translation of preclinical research [172].

Assessing endogenous transporter function in clinical settings particularly presents challenges due to the absence of reliable biomarkers. Unlike drug–drug interaction studies, which leverage pharmacokinetic measurements to evaluate transporter activity, the physiological roles of transporters lack direct and easily measurable indicators. This deficiency complicates efforts to correlate transporter function with systemic physiological effects, limiting the ability to assess their contribution to disease mechanisms and therapeutic responses [173]. Additionally, genetic polymorphisms that modulate transporter activity do not always translate directly to functional consequences [174,175], making it difficult to establish clear associations with disease states or metabolic variations.

Despite the numerous challenges associated with studying endogenous functions of drug transporters, advancements in technology and novel research strategies are paving the way for new discoveries. Together, these efforts have the potential to overcome the experimental and translational barriers that currently limit our understanding of transporter biology, unlocking their potential as therapeutic and diagnostic targets.

## 6. Future Research Directions

Recent advancements in experimental methodologies have enhanced our understanding of transporter expression, function, and their roles in pathophysiology. A prime example is the emergence of single-cell and spatial transcriptomics, which have become powerful tools for mapping transporter expression across distinct cell populations and microenvironments. These approaches enable the identification of novel cell states and interactions, providing deeper insights into transporter-mediated mechanisms [176]. Spatial transcriptomics links gene expression to anatomical context, enabling the mapping of transporter expression across specific tissue regions and cell types. It also enables assessment of how these spatial patterns change in disease-associated microenvironments [177]. This has facilitated the discovery of transcriptionally distinct cell states, such as inflammatory epithelial subtypes or reprogrammed stromal populations, that exhibit unique transporter profiles in response to pathological cues [178]. Additionally, the combined application of single-cell and spatial transcriptomics has led to the identification of epithelial-immune interactions in kidney injury, elucidating the role of transporters in tissue-specific pathophysiology [179]. By capturing the spatial organization and dynamic regulation of transporters, these methodologies offer unprecedented opportunities to delineate disease-specific alterations.

Omics-based methodologies, including transcriptomics, proteomics, metabolomics, and lipidomics, have become indispensable in drug transporter research, offering a systems-level perspective on transporter function and its broader physiological impact. These approaches link genetic variations to metabolite regulation and transporter-substrate interactions, shedding light on metabolic pathways involved in cellular homeostasis and disease progression [180,181]. By integrating metabolomic and transcriptomic profiling, studies have uncovered adaptive metabolic shifts in transporter-deficient models, revealing compensatory mechanisms that help maintain homeostasis [182]. Additionally, omics-based analyses have provided insights into the interplay between transporters, microbiome dynamics, and inter-organ communication, emphasizing their role in metabolic regulation [20,22]. Coupling multi-omics with functional genomics and computational modeling holds significant promise for refining transporter research. This convergence will not only enhance biomarker discovery but also facilitate the development of targeted therapies aimed at improving drug efficacy while minimizing adverse effects [183,184].

Gene editing technologies, particularly CRISPR-based approaches, are transforming drug transporter research by enabling precise functional characterization of transporter genes. These methodologies facilitate the generation of knockout and gain-of-function models, making it possible to dissect transporter functions, distinguish primary effects from secondary metabolic adaptations, and refine our understanding of their roles in drug disposition and disease [15,185]. High-throughput CRISPR screening has uncovered key transporters involved in drug resistance, metabolic adaptation, and environmental stress responses, advancing understanding of their regulatory networks [186,187]. Additionally, the role of efflux transporters such as MRP1 in modulating drug sensitivity has been increasingly recognized, underscoring the need for novel strategies to overcome transporter-mediated resistance and improve therapeutic efficacy [188]. The development of humanized models incorporating human transporter genes into both in vitro and in vivo systems enhances translational relevance, allowing for more accurate predictions of transporter function in clinical settings.

Organoid and microfluidic models also emerge as transformative tools in drug transporter research, offering physiologically relevant platforms for studying transporter function, drug disposition, and toxicity in a controlled microenvironment. Kidney proximal tubule-on-chip systems and human-induced pluripotent stem cell (hiPSC)-derived kidney organoids have enabled functional characterization of renal transporters, improving predictions of drug uptake, clearance, and nephrotoxicity [189,190]. These models integrate microfluidic flow dynamics, mimicking in vivo-like shear stress and transporter-mediated solute exchange, thereby enhancing their translational relevance [191,192]. Biosensor-equipped organoids represent an advanced iteration of organ-on-a-chip technologies, integrating real-time molecular sensing with physiologically relevant tissue architectures. This fusion enables dynamic assessment of transporter activity and ATP/ADP energy dynamics, facilitating high-throughput screening for drug-induced nephrotoxicity and transporter-drug interactions [193]. Furthermore, the application of human organoid-based systems in predictive toxicology has significantly improved preclinical drug evaluation by recapitulating patient-specific transporter function and variability [194]. As these platforms continue to evolve, their combination with CRISPR-based gene editing, multi-omics profiling, and computational modeling will further refine our understanding of transporter-mediated biological processes and drug responses, ultimately shaping the next generation of precision medicine and accelerating the development of safer, more effective therapeutics.

## 7. Concluding Remarks

Understanding the diverse roles of drug transporters beyond drug disposition and pharmacokinetics is essential for both advancing basic research and clinical applications. These transporters not only influence drug efficacy and safety but also play key roles in physiological regulation and disease pathology. As emerging evidence continues to highlight their contributions to metabolic homeostasis, immune responses, and cellular detoxification, a more comprehensive approach to transporter research is needed. Future studies should aim to further elucidate the endogenous functions of these proteins and their impact on disease progression. Expanding our knowledge of transporter mechanisms could pave the way to identifying novel therapeutic strategies, including targeted modulation of transporter activity in specific disease contexts. Additionally, integrating transporter research into clinical practice may improve drug development and precision medicine, ultimately enhancing patient outcomes. In embracing the full biological and clinical significance of drug transporters, we move closer to unlocking their potential as both biomarkers and therapeutic targets in the next generation of biomedical innovation.
